# Role of point-of-care ultrasound during the COVID-19 pandemic: our recommendations in the management of dialytic patients

**DOI:** 10.1186/s13089-020-00177-4

**Published:** 2020-06-03

**Authors:** Ana Luisa Silveira Vieira, José Muniz Pazeli Júnior, Marcus Gomes Bastos

**Affiliations:** 1 Department of Nephrology, Santa Casa de Misericórdia de Barbacena and University of Medicine of Barbacena Department of Point of Care Ultrasound, Minas Gerais, Brazil; 2 Department of Nephrology and Intensive Care Medicine, Santa Casa de Misericórdia de Barbacena and University of Medicine of Barbacena Department of Point of Care Ultrasound, Minas Gerais, Brazil; 3 Federal University of Juiz de Fora, Minas Gerais, Brazil; 4 Faculty at School of Medicine, UNIFAGOC and SUPREMA, Minas Gerais, Brazil

**Keywords:** COVID-19, SARS-CoV-2, Dialysis, COVID-19 pandemic, Ultrasound

## Abstract

COVID-19 is a viral disease due to the infection of the novel Corona virus SARS-CoV-2, that has rapidly spread in many countries until the World Health Organization declared the pandemic from March 11, 2020. Elderly patients and those affected by hypertension, diabetes mellitus, and chronic pulmonary and cardiovascular conditions are more susceptible to present more severe forms of COVID-19. These conditions are often represented in dialytic renal end-stage patients. Moreover, dialysis patients are more vulnerable to infection due to suppression of the immune system. Growing evidences, although still supported by few publications, are showing the potential utility of ultrasound in patients with COVID-19. In this review, we share our experience in using point-of-care ultrasound, particularly lung ultrasound, to indicate the probability of COVID-19 in patients with end-stage renal disease treated by hemodialysis. We also propose recommendations for the application of lung ultrasound, focused echocardiography and inferior vena cava ultrasound in the management of patients in hemodialysis.

## Introduction

The world is currently facing a pandemic disease caused by a novel coronavirus (SARS-CoV-2) and called COVID-19 (COronaVIrus Disease-19, described in 2019). This viral infection showed rapid dissemination and potential harm due to complications in the respiratory system characterized by a severe form of pneumonia with respiratory failure. Only a minority of patients are at risk of developing the severe form of the disease, but the extremely high number of infected patients is causing a surge that is creating a serious crisis even in the most advanced medical organizations [1]. The big challenge is to recognize, efficiently and quickly, those patients with COVID-19 pneumonia who need isolation, hospitalization and intensive care management.

The Chinese experience shows that clinical symptoms at presentation are not able to predict the severity of the disease and that transmission is frequent from asymptomatic infected subjects [2]. Chest radiography, despite its wide availability and low cost, has low diagnostic sensitivity for pneumonia in cases of COVID-19 [3]. On the other hand, lung computed tomography (CT) is considered to be the best image modality for early identification of pulmonary involvement in patients infected with SARS-CoV-2 [1]. In a recent study, the sensitivity of CT was reported to be superior to reverse transcription polymerase chain reaction (RT-PCR) (98% vs. 71%, respectively, *p* < 0.001) in the diagnosis of COVID-19 [4]. However, a role of CT imaging in the diagnosis of COVID-19 is ambiguous and this latter study has been heavily criticized [5]. Moreover, it is difficult to imagine a scenario where CT is systematically performed in all suspected cases of COVID-19 because of costs, time consumption, exposure to radiation, possibility of cross-infections, and even non-availability in scarce resource areas.

Patients with end-stage renal disease (ESRD) are at increased risk of infection with SARS-CoV-2 virus. Reports from health services around the world have indicated that patients with diabetes mellitus and hypertension, two of the main causes of ESRD worldwide, and also advanced age and cardiovascular complications, two frequent accompanying conditions in dialysis patients, are more susceptible to SARS-CoV-2 infection and more prone to develop severe COVID-19 pneumonia, eventually requiring intensive care treatment [2, 6, 7]. Moreover, the logistical characteristics of hemodialytic procedures, performed three times a week in closed areas to group of patients, are at increased risk of disease transmission [8]. So far, epidemiological data in patients on ambulatorial hemodialysis with COVID-19 are limited. During the outbreak in a hemodialysis unit of Wuhan, it was reported that 37 out of 230 patients (16.1%) and 4 out of 33 staff personal (12.1%) were diagnosed with COVID-19 [7]. During the 33 days of follow-up, seven of those patients died.

Lung ultrasound (LUS) shows superior sensitivity when compared to X-ray in patients with acute respiratory failure [9]. Moreover, LUS has been experienced during the Chinese outbreak of COVID-19 [3, 10]. Nonetheless, LUS may be a more feasible and practical alternative since it can be quickly done at the bedside, is repeatable and reduces the possibility of cross-infections. In Italy, LUS has been proposed as an alternative to CT for suspected cases of COVID-19 [11–14].

Based on our experience and supported by the existing literature, we suggest the use of LUS in dialysis patients to allow the early recognition of the disease as well as to indicate the severity of pulmonary involvement, allowing a safer allocation of dialysis patients during the COVID-19 pandemic.

### The special role of LUS during the COVID-19 pandemic

The role of LUS to evaluate several respiratory conditions is nowadays widely documented [9]. The lesions observed in CT scan of patients with COVID-19 are generally peripheral and subpleural, perhaps due to the fact that SARS-CoV-2 is a novel virus with an average diameter of only about 120 nm, which allows it to be easily inhaled down to the peripheric airways and alveoli without immune barrier [10].

LUS is an image method that classically allows the evaluation of the pulmonary periphery which makes it an excellent technique in the assessment of COVID-19. The same physician in charge of the patient can obtain pulmonary images by LUS performed at bedside, thus minimizing the number of health professionals potentially exposed to the virus. This is a crucial point, considering that data from Italy and Spain, two of countries with the highest rate of COVID-19, show that approximately 9 to 12% of health workers became infected and were quarantined [15].

The technology of modern ultrasound machines (portable and ultra-portable) currently available has many favorable characteristics. The easy portability at the bedside, the long battery life and the possibility to be connected to smartphones and tablets make them suitable even in remote areas. The small dimension is also a favorable characteristic to allow an easy procedure for disinfection, which is crucial in the context of the current pandemic. The application of LUS by portable machines allows the examination without the need to move the patient to radiology facilities, which would increase the risk of cross-infections. Another important advantage of modern technology is connectivity that allows the possibility of image sharing through social media and other internet systems, making telemedicine a possible help to local medical staffs. However, not always hand-held machines are available in the medical units. LUS is becoming critical in the front door of this COVID-19 outbreak. Thus, any ultrasound machine should be used based on the availability. Carriable ultrasound machines can be dedicated to specific areas where patients suspected for COVID-19 are examined. Anyway, we recommend an accurate disinfection of the probes after each exam using alcohol solutions to avoid contamination. In case of use of the ultrasound machine during invasive procedures at risk of aerosol dispersion, we also recommend using transparent covers on the keyboard and a full disinfection of the screen and other parts at the end of the maneuver.

### Basic principles of LUS

LUS is a relatively new technique that has been used increasingly since the pioneering work of Lichtenstein and Axler, published in the early 90s [16]. Nowadays, LUS is frequently used in the emergency departments and intensive care units in the diagnosis of several lung diseases [17]. More recently, nephrologists also discovered LUS as a great tool to evaluate their dialytic patients [18, 19].

**Fig. 1:**
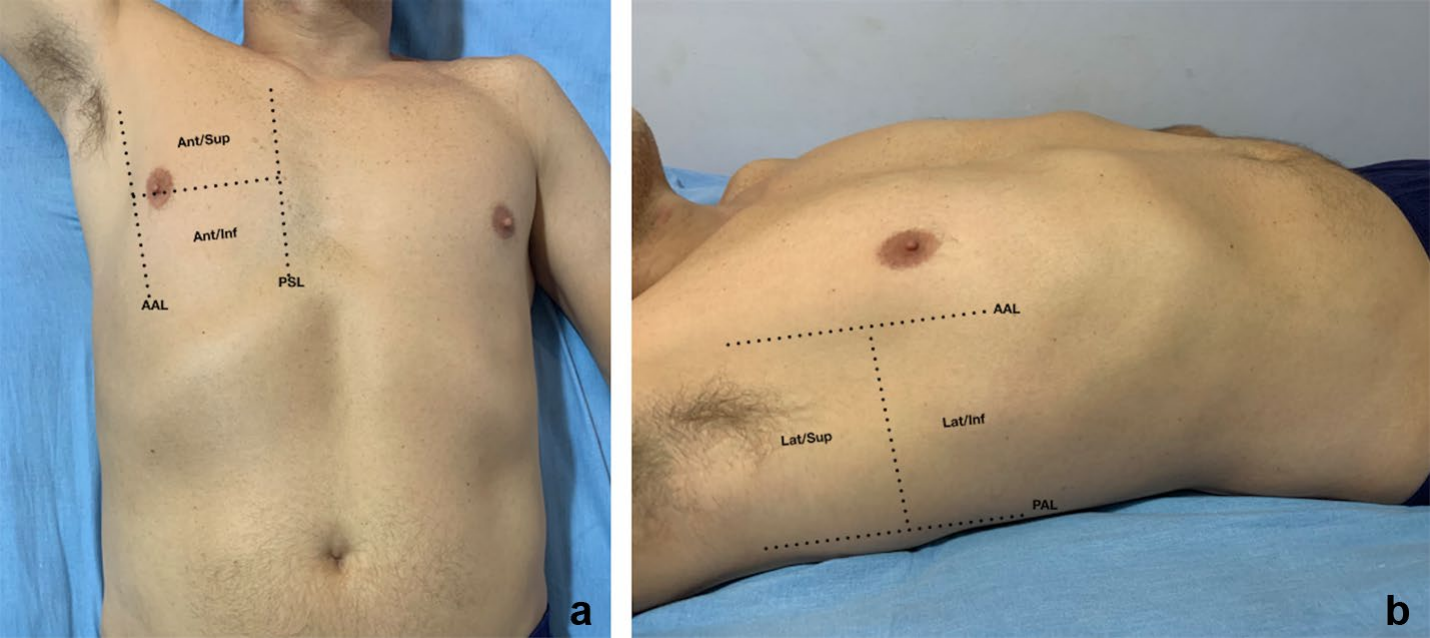
Anterior and lateral chest. **a** Anterior chest is delimited by parasternal line (PSL) and anterior axillary line (AAL) and divided in superior (ant/sup) and inferior (ant/inf). **b** Lateral chest is delimited by anterior axillary line (AAL) and posterior axillary line (PAL) and divided in superior (lat/sup) and inferior (lat/inf)

**Fig. 2:**
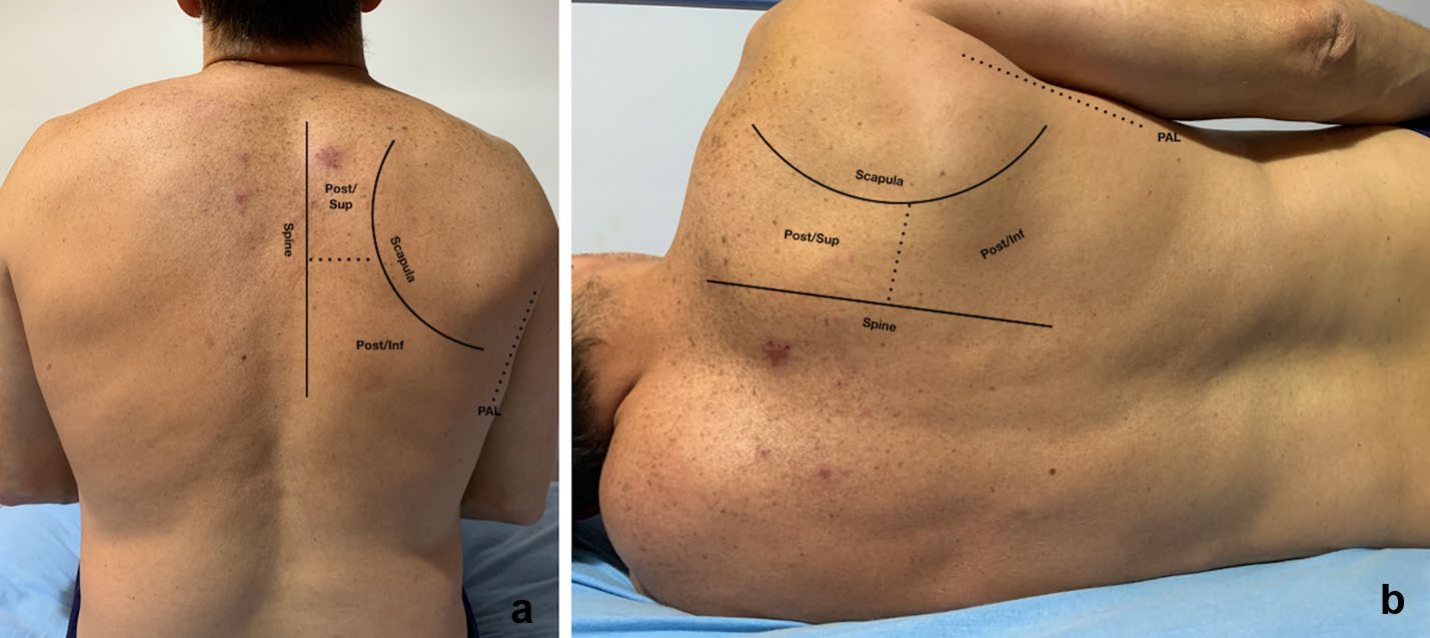
Posterior chest in sitting and lateral decubitus positions. Posterior chest is delimited by the spine and posterior axillary line (PAL) and divided in superior (post/sup) and inferior (post/inf). **a** Patient in sitting position. **b** Patient in lateral decubitus position

The images obtained with LUS most of the time are not anatomical. They are based on artifacts generated by the interaction of the ultrasound beam with the acoustic interface between the tissues of the chest wall and the air in the alveoli distributed in the pulmonary surface. Indeed, LUS can only study lung diseases that abut the pleura [16, 20]. Patients can be examined either in supine/semi-recumbent (with arm abducted over the head) or in sitting position, as similarly done during the standard physical examination [21]. Each hemithorax is generally divided into three areas: anterior (between parasternal and anterior axillary lines), lateral (between anterior and posterior axillary lines) (Fig. 1) and posterior (between posterior axillary line and the spine) (Fig. 2). Each area is further divided into superior and inferior [9]. In the case of critical care patients who cannot collaborate and lie down in the supine position, the dorsal region of both lungs can be assessed by slightly moving the patient in lateral decubitus [22]. The lungs can be scanned using a high-frequency probe (linear) or by a low-frequency probe (convex, microconvex and phased array). The higher the frequency, the greater is the image resolution, even though penetration is reduced. On the other hand, low-frequency transducers can reach greater depths, but at the expense of a lower image resolution. In the context of this pandemic, we recommend using a probe with a large surface of emission and a technique for LUS that allows a quick examination of the whole chest surface. Also, the probe should transmit at a low frequency to allow enough penetration and evaluate pleural surface and vertical artifacts at the same time. This description corresponds to the convex probe. The transducer should be positioned longitudinally to the chest and perpendicular to the ribs. On occasion, the probe can be turned to obtain an oblique scan along the intercostal space to visualize more in detail the LUS pattern in a wider portion of the pleural surface. LUS allows a dynamic examination in real time and the transducer must be handled with continuous slow movements of tilting, sliding and rotating to optimize the image. During the insonation, it is also helpful to adjust the image gain and depth (around 8 to 10 cm) and position the focus (when available) at the level of the pleural line. As LUS is based on artifacts, it is important to disable the “Multi Beam”, “Tissue Harmonic Imaging” features and filters that optimize the real images and decrease artifacts [20].

### Lung patterns

**Fig. 3:**
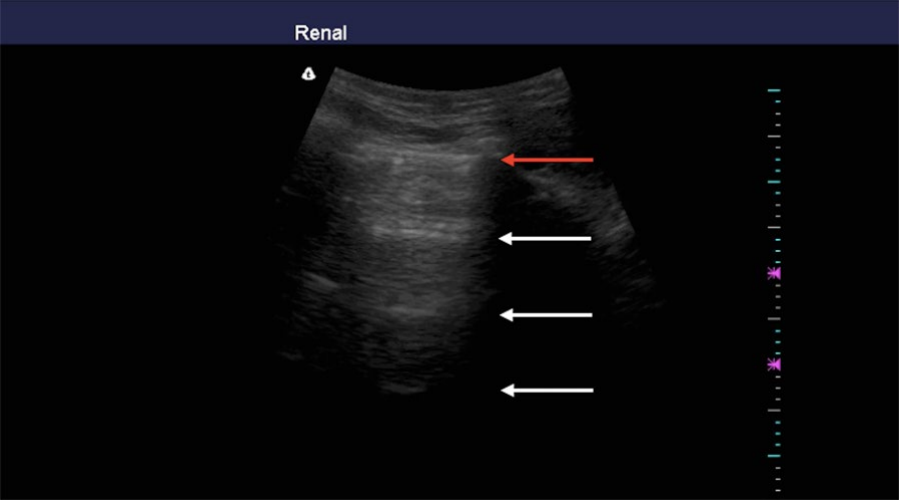
Sonographic appearance of an aerated lung scan in longitudinal view. Red arrow indicates pleural line; white arrows indicate A-lines

LUS is a technique mainly based on the visualization of artifacts originating by the acoustic mismatch between tissue and air in the surface of the lung [12]. Normally, in the periphery of the lung, there is basically alveoli-filled with air. When the ultrasound beam encounters the interface between two structures with opposite values of acoustic impedance, it is completely reflected back to the transducer. Thus, during the chest insonation, the ultrasound beam will pass through skin, subcutaneous tissue, muscles, until it reaches the pleura. This latter corresponds to the interface where the maximum acoustic tissue/air mismatch is encountered and will be visualized as an echoic horizontal linear artifact, the pleural line. Below the pleural line, the examiner may see echoes that reproduce the pleural line at regular equidistant intervals echoing more times the distance between the probe and the pleura. These horizontal artifacts, indicated as A-lines, are visualized when the lung is well aerated (Fig. 3) (Additional file 1) [23].

**Fig. 4:**
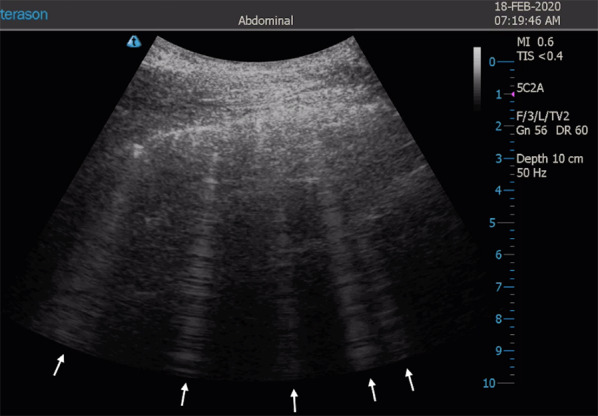
Sonographic appearance of B-lines in oblique view. White arrows indicate multiple B-lines

**Fig. 5:**
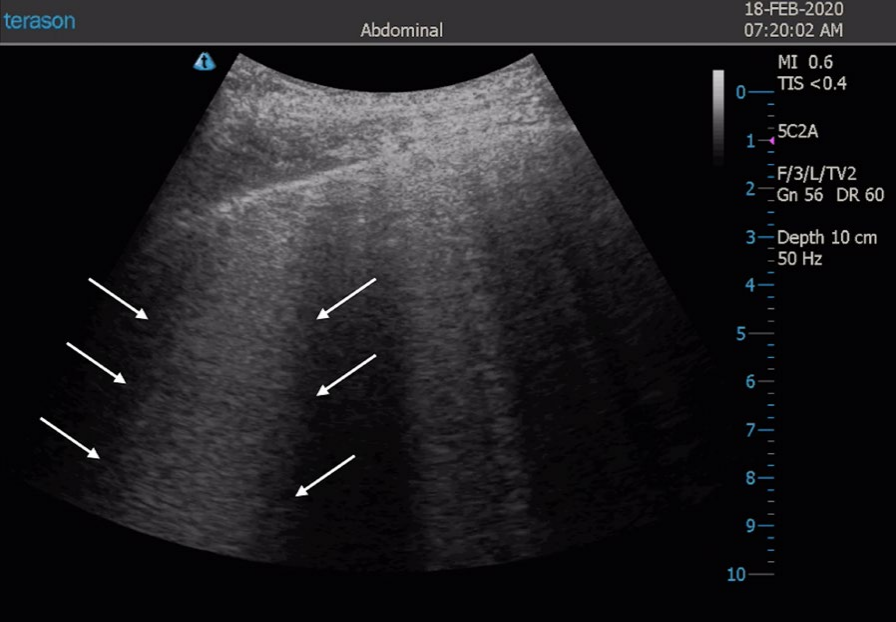
Sonographic appearance of coalescent B-lines. White arrows indicate area with coalescent B-lines, called white lung

**Fig. 6:**
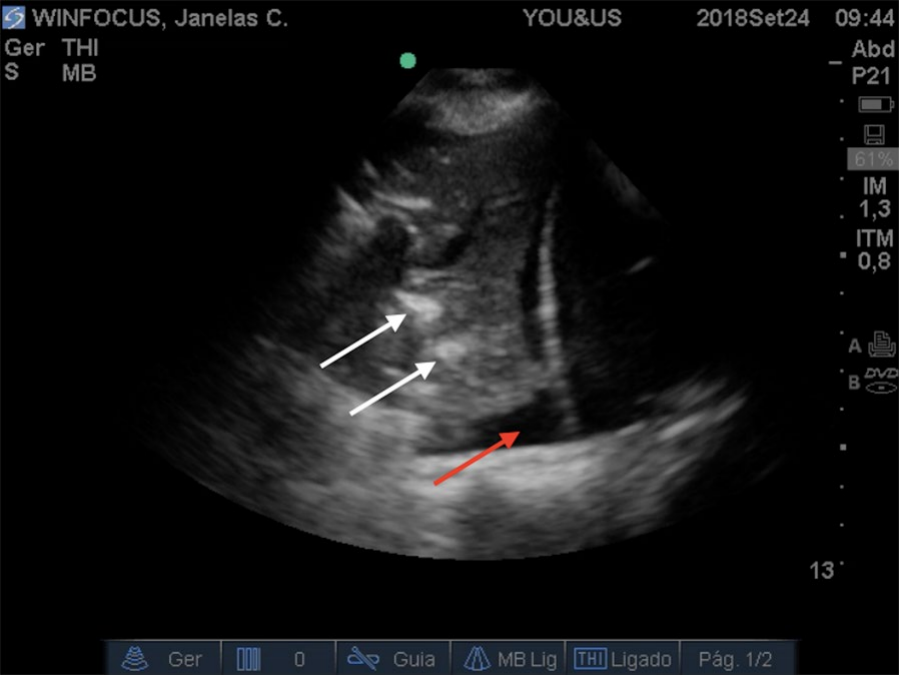
Sonographic features of lung consolidation. The echo-texture of lung becomes similar to the liver. White arrows indicate air bronchograms. Red arrow indicates pleural effusion

Accumulation of subpleural interstitial exudate, transudate, collagen, and blood causes loss of aeration and changes the air–liquid balance. Also, the fibrotic thickenings of the interlobular septa create a similar deaeration. In these pathologic conditions, the pulmonary surface will not be a strong reflector of the ultrasound beams. The first grade of loss of aeration causes a partial penetration of the ultrasound creating vertical artifacts on the images displayed on the screen. Nowadays, these artifacts are called B-lines [12]. B-lines are defined as discrete laser-like vertical hyperechoic reverberation artifacts that arise from the pleural line, extend to the bottom of the screen without fading, and move synchronously with lung sliding [24] (Fig. 4) (Additional file 2). When the accumulation of fluid raises, the B-lines proportionally increase in number and intensity until tend to coalesce, originating a pattern called white lung [23] (Fig. 5) (Additional file 3). In this progression, the final step of loss of aeration and increase in density gives the pattern of a consolidated lung parenchyma [21] (Fig. 6) (Additional file 4).

### How to interpret the findings of LUS in dialytic patients suspicious for COVID-19

LUS allows a first screening of patients admitted at the dialysis unit presenting symptoms that raise suspicion of COVID-19. It can help triaging between low-risk patients (patients clinically stable who do not present pathologic LUS changes) and those who show signs of pneumonia and high risk for severe forms of the disease. Thus, LUS allows us to diagnose pneumonia in COVID-19 and differentiate the severity of the disease at presentation, useful to select who requires hospitalization and limit unnecessary nosocomial exposure. However, it is important to highlight that in case of abnormal LUS pattern suggestive of viral pneumonia, ruling out alternative conditions may represent a challenge. Indeed, B-lines and consolidations are not enough specific and cannot easily differentiate hydrostatic/cardiogenic edema or other pathologic conditions [14].

**Fig. 7:**
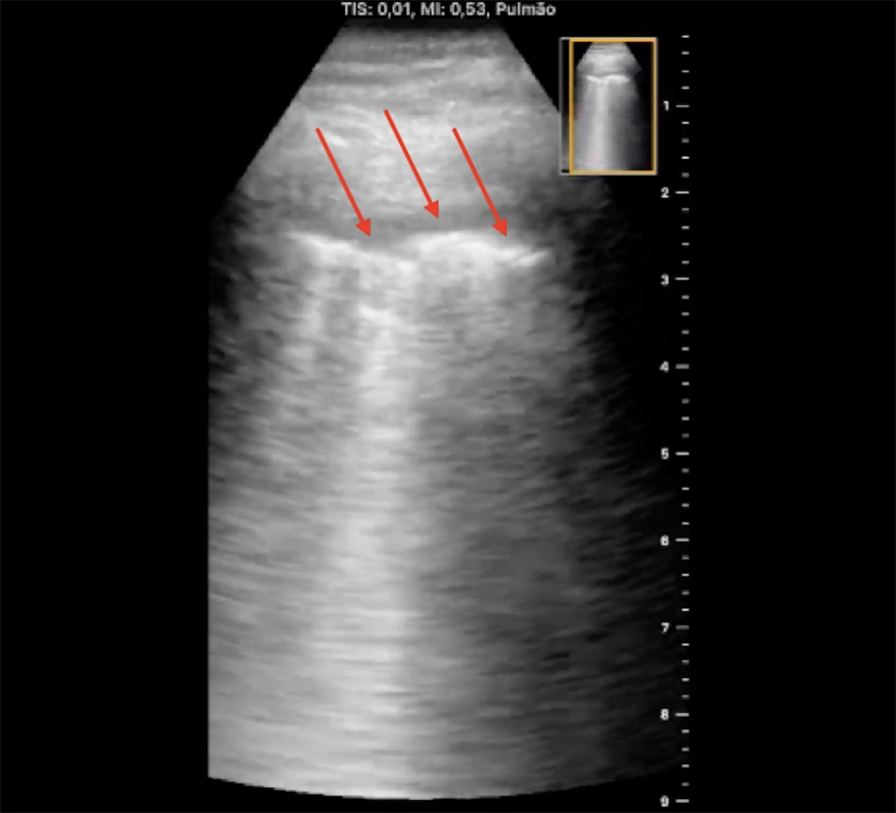
Sonographic appearance of pleural irregularities in a COVID-19 patient. Red arrows indicate fragmented and irregular pleural line

The interstitial pneumonia in COVID-19 is typically peripheral, bilateral, diffuse but asymmetric [14]. For this reason, the LUS exam in the suspected patient must be performed all over the chest surface [10]. The typical patterns detected by LUS in patients with COVID-19 pneumonia are characterized by B-lines in different forms, both separated and coalescent, an irregular and/or fragmented pleural line, peripheral small consolidations, and large consolidations with dynamic air bronchograms [3] (Fig. 7) (Additional file 5). These patterns are usually intercalated with “spared areas” (A-lines) [13]. A large pleural effusion is not a common finding [3]. Detection of the typical signs, described above combined in a pattern with a typical bilateral and patchy distribution, raises the high probability of the presence of the disease in a symptomatic patient.

**Fig. 8:**
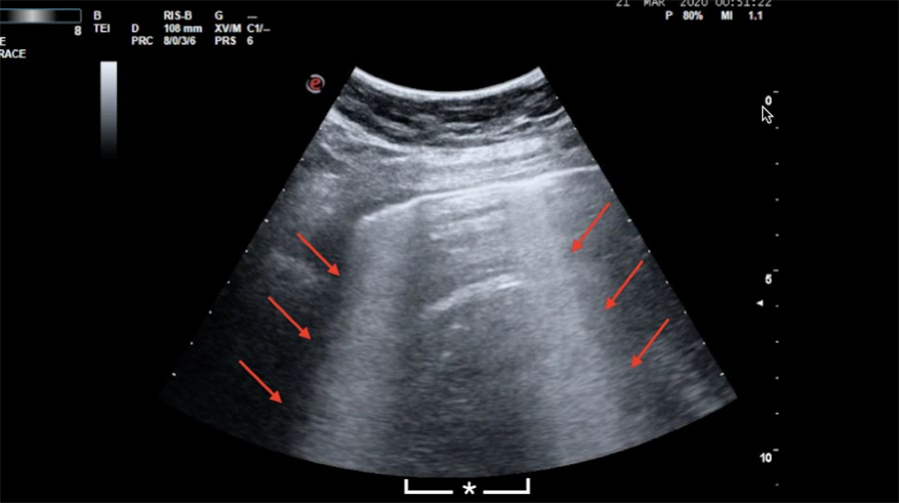
Sonographic appearance of “light beam” artifact. Red arrows indicate band-form signs arising from a large portion of a regular pleural, called “light beam”. The asterisk indicates the spared area (A-lines). Image kindly provided by Dr. Giovanni Volpicelli

However, in dialytic patients the interpretation of the artifacts is more challenging, due to the fact that these frequently are affected by cardiovascular complications and quite frequently present a status of hypervolemia [25]. It is especially critical to differentiate the characteristics of the B-lines in dialytic patients admitted with dyspnea, since these artifacts are the most frequent findings in COVID-19 pneumonia and also typically found in hypervolemic patients with lung congestion. B-lines due to hypervolemia are symmetrically distributed in both lungs and initially well separated. The symmetric distribution is gravity related and the anterior superior areas are the last part of the lung to be involved in the process of progression of the severity of lung congestion. Thus, correlation of the severity of the B-lines representation and the severity of the symptoms of the patient becomes critical. Furthermore, the pleural line is usually diffusely regular, and lung consolidations are not detected. Another differential characteristic is that the pleural effusion is a common finding in hypervolemia. On the other hand, although B-lines due to COVID-19 are also typically bilateral, they have a different distribution. In pneumonia during the course of COVID-19, it is typical the visualization of clusters of B-lines, both in separated and coalescent forms. Moreover, other authors described an intense “shining band-form artifact spreading down from a large portion of a regular pleura, often appearing and disappearing with an on–off effect in the context of a normal A-lines lung pattern visible on the background” [14] (Fig. 8). This sign has been called “light beam” artifact (Additional file 6). This band may also originate from an irregular pleural line or from small subpleural consolidations. The “light beam” is not only observed in COVID-19 pneumonia but it is highly frequent in this disease. The combination of the light beam with the other artifacts in clusters alternating abruptly with spared A-lines areas, with the typical patchy distribution, gives the specificity to LUS for the diagnosis of COVID-19 pneumonia [13, 14]. This is particularly effective when LUS is applied in patients presenting with the typical symptoms of COVID-19, in this specific moment of outbreak with a high prevalence of the disease.

If the B-lines pattern that can be observed by LUS in dialytic patients does not fully respect the typical characteristics of a COVID-19 pneumonia and cannot allow a definitive conclusion, we suggest to extend the ultrasound scan to the heart and the inferior vena cava (IVC). ESRD patients frequently present cardiovascular complications, giving combined or separated diastolic and systolic dysfunctions. These dysfunctions frequently are associated and even favor hypervolemia, and may easily result in pulmonary congestion [26]. Fluid congestion of the pulmonary interstitium is represented with high sensitivity by multiple and diffuse B-lines at LUS. Not only LUS is highly sensitive in the detection of pulmonary congestion, but also it can indicate the effectiveness of a treatment that reduces the congestion. Thus, if monitoring the patients during the dialysis session the number of B-lines decreases after the ultrafiltration, it is a reliable indicator that the origin of the LUS pattern is hypervolemia and not the infection [27].

The ultrasound evaluation of the diameter and the respiro-phasic change of IVC can also be helpful in a scenario where differential interpretation of B-lines is a challenge. A collapsing or close to collapsing IVC before the dialysis session is not compatible with a condition of hypervolemia. On the other hand, a plethoric IVC shortly after dialysis implies a still filled intravascular space. However, the ultrasound examination of the IVC poses always the necessity to consider possible confounding factors. It is essential to consider the clinical settings. For instance, there are situations when the decrease of IVC diameter is not related to progression to hypovolemia, but may be linked to increased abdominal pressure, an abnormal inspiratory effort, and others. At the same time, situations revealing increase in the IVC diameter may be not necessarily related to hypervolemia, like it is observed in some specific cardiac conditions determining an obstacle to the venous return or in pulmonary conditions with alveolar hyperinflation, and so on [28].

### Using ultrasound in dialysis patients during COVID-19 pandemic

As seen previously in this article, it is clear that dialysis patients are at a greater risk of developing the most severe forms of COVID-19, in addition to being potential vectors of the disease; since due to the specificities of their treatment, they cannot fully adhere to the social distancing, recommended by the World Health Organization [29].

**Fig. 9:**
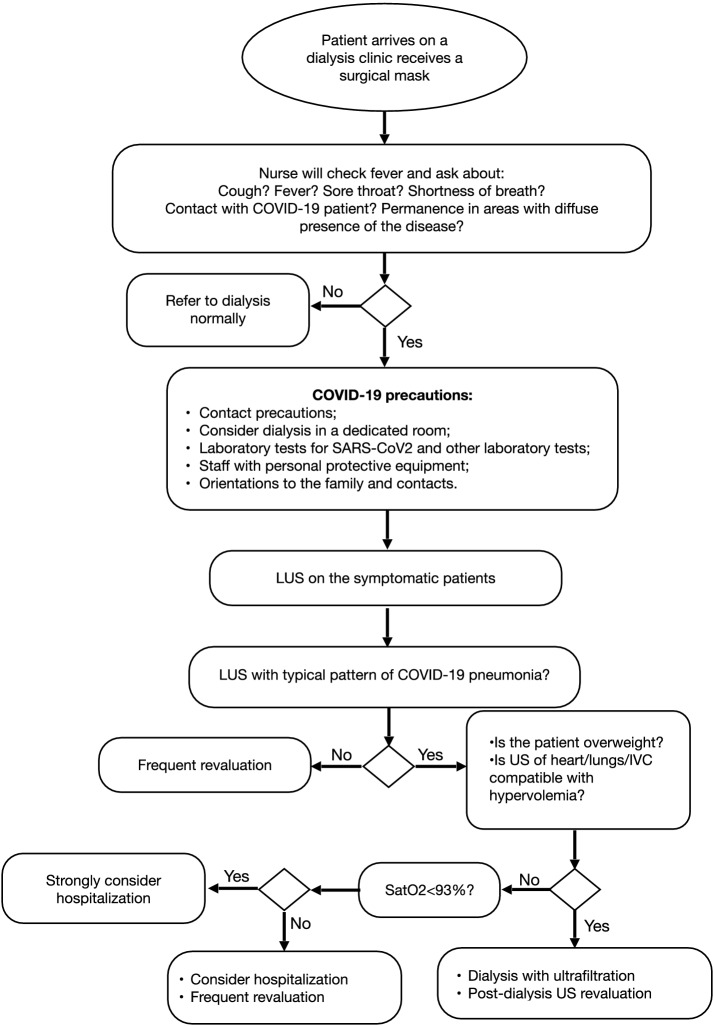
Flowchart of our recommendations for management of dialysis patients in COVID-19 pandemic

Based on current evidence and our experience with point-of-care ultrasound in nephrology, we suggest the following recommendations in the management of dialytic patients during the pandemic (Fig. 9):

1. The creation of different allocations, whenever possible, for patients who arrive and leave the clinic, thus reducing contacts between patients;
2. Immediate supply of surgical masks as soon as patients arrive to the unit for the dialysis session. The mask must be kept by the patient for the entire stay in the clinic. During triage, a health professional, also wearing a surgical mask, should investigate about any suspected symptom of COVID-19 infection, such as fever, cough and dyspnea, and possibility of any contact with confirmed cases of COVID-19 or permanence in areas with diffuse presence of the disease. In addition, the temperature must be checked using an infrared thermometer, minimizing contacts with the patient;
3. If the patient is asymptomatic and does not confirm any contact with confirmed cases, the patient can be referred to his/her dialysis session, as usual;
4. If the patient is asymptomatic but confirms any contact with positive COVID-19 cases or provenience from an area of strong epidemic, he/she should preferably be referred to an isolated room dedicated to suspected cases, where patients remain separated with a minimum distance of 1.82 m [8]. In this room, particular care should be reserved to allow isolation from contact and protection of the dialysis personnel, who should wear full protective gear such as waterproof disposable gown, cap, gloves, face shield, and at least N95 face mask, and proceed with more rigorous cleaning and disinfection. Complementary tests for virus identification, and RT-PCR for SARS-CoV-2 can also be ordered, if available. Until the result of the test is obtained, all the family members should be alerted and the patient should maintain isolation for at least 14 days [29];
5. If the patient presents with signs and symptoms of the disease, all the previous measures should not only be adopted but the patient should also be studied by LUS at triage. The signs characteristic of the disease should be investigated, with a particular focus on the presence, characteristics and distribution of B-lines;
6. If the patient shows the typical LUS pattern of COVID-19 pneumonia without doubts about the alternative possibility of hydrostatic pulmonary edema, the next step is to assess the peripheral oxygen saturation. If saturation is below 93%, hospital admission should be strongly considered. If saturation is above 93%, the cost–benefit ratio of hospitalization should be considered. Other factors, like adequate family support, comorbidities and socio-economic conditions, are important conditions that need to be investigated and considered. In the case a patient with positive signs of COVID-19 pneumonia is discharged to home, frequent clinical re-assessment should be programmed;
7. If the patient shows a pattern of B-lines not typical and hydrostatic edema remains a possible alternative, ideally, a more detailed ultrasound assessment should be performed, by extending the examination to the heart and IVC. If the ultrasound findings are compatible with hydrostatic edema, the patient should be submitted to dialysis with ultrafiltration and reassessed clinically and with ultrasound at the end of the procedure. If the clinical status and the LUS pattern improve significantly in terms of resolution of dyspnea and reduction in the number of B-lines, the possibility that the LUS pattern observed at triage was secondary to hypervolemia is reinforced. If there is no change in clinical and LUS parameters, the possibility of an infective origin of B-lines increases;
8. In the context of the pandemic that we are experiencing, it is essential to pay attention to measures of individual protection and the accurate cleaning and disinfection of the device and transducers after their use. The examiner must wear an apron, gloves (three pairs), glasses and N95 mask. The patient must wear a surgical mask during the examination [30]. To avoid contamination of the US device, it is recommended to cover the equipment keyboard with transparent plastic films to be changed after each use. It is also recommended to clean the device with gauze or similar material soaked in 70% alcohol and the transducers according to the manufacturer’s guidelines (in general, compounds based on quaternary ammonia are recommended). The first pair of gloves should be removed after examining the patient, the second pair after disinfecting the equipment and the third pair together with the removal of the garment [31].

## Conclusion

The world is witnessing a pandemic with disastrous effects due to the rapid spread of the SARS-CoV-2 virus and the huge demand for resources needed to contain COVID-19. In this context, LUS performed by the attending physician represents a safe, inexpensive, easily reproducible technique with a great potential in allowing to differentiate patients with signs of COVID-19 pneumonia. The screening performed by the LUS at the bedside allows defining the immediate management of dialysis patients, seen in dialysis units, where other propaedeutic resources are often not available. A strategy based on triaging suspected patients for COVID-19 pneumonia is potentially useful to prevent the dissemination of the virus and also to impact positively the prognosis of vulnerable dialytic patients.

## Supplementary information

**Additional file 1: Video 1.** A-lines. Longitudinal scan on the anterior chest of a patient with aerated lung. The video demonstrates the A-lines.

**Additional file 2: Video 2.** Multiple B-lines. Oblique scan on the anterior chest of a hypervolemic patient with multiple (but not coalescent) B-lines.

**Additional file 3: Video 3.** Coalescent B-lines. Oblique scan on the lateral chest of a hypervolemic patient with coalescent B-lines.

**Additional file 4: Video 4.** Lung Consolidation and Pleural effusion. Scan of the right lung base of a patient with pneumonia. The video shows lung consolidation with dynamic air bronchogram and pleural effusion.

**Additional file 5: Video 5.** Pleural irregularity. Scan of a patient with COVID-19 performed by a hand-held device showing pleural irregularities.

**Additional file 6: Video 6. “**Light Beam” sign. Oblique scan of a patient with COVID-19 with “light beam” signs with on–off effect and separated by spared areas (A-lines).

## Data Availability

Availability of data is not applicable to this article.

## Abbreviations

COVID-19: Corona Virus Disease-19
CT: Computed tomography
ESRD: End-stage renal disease
HD: Hemodialysis
IVC: Inferior vena cava
LUS: Lung ultrasound
RT-PCR: Reverse transcription polymerase chain reaction
SARS-CoV-2: Severe Acute Respiratory Syndrome Corona Virus
US: Ultrasound
